# A Range-Expanding Shrub Species Alters Plant Phenological Response to Experimental Warming

**DOI:** 10.1371/journal.pone.0139029

**Published:** 2015-09-24

**Authors:** Christopher W. Kopp, Elsa E. Cleland

**Affiliations:** 1 The Biodiversity Research Centre, The University of British Columbia, Vancouver, Canada; 2 Ecology, Behavior & Evolution Section, Division of Biological Sciences, University of California San Diego, San Diego, United States of America; INRA - University of Bordeaux, FRANCE

## Abstract

Shifts in plant species phenology (the timing of life-history events such as flowering) have been observed worldwide in concert with rising global temperatures. While most species display earlier phenology with warming, there is large variation among, and even within, species in phenological sensitivity to rising temperatures. Other indirect effects of climate change, such as shifting species composition and altered species interactions, may also be contributing to shifting plant phenology. Here, we describe how experimental warming and the presence of a range-expanding species, sagebrush (*Artemisia rothrockii*), interact to influence the flowering phenology (day of first and peak flowering) and production (number of flowers) of an alpine cushion plant, *Trifolium andersonii*, in California’s White Mountains. Both first flowering and peak flowering were strongly accelerated by warming, but not when sagebrush was present. Warming significantly increased flower production of *T*. *andersonii*, but less so in the presence of sagebrush. A shading treatment delayed phenology and lowered flower production, suggesting that shading may be the mechanism by which sagebrush presence delayed flowering of the understory species. This study demonstrates that species interactions can modify phenological responses to climate change, and suggests that indirect effects of rising temperatures arising from shifting species ranges and altered species interactions may even exceed the direct effects of rising temperatures on phenology.

## Introduction

Along with range shifts [1–4], earlier spring phenology (i.e., the timing of biological events such as leaf emergence or flowering) observed worldwide [5–8] provides some of the best evidence that plant species are already responding to climate change [1,2,7,9–11]. While advanced spring phenology is often observed in response to warming, there is significant variation among species in the magnitude and even direction of phenological responses [5–8]. Along with temperature having a high degree of influence on phenological sensitivity [12], species interactions can also influence phenology [13], and hence may contribute to the observed variation. Most attention has focused on biotic interactions such as pollination [14,15] or trophic interactions [1,2,16] for which differing phenological sensitivities among interacting species could result in mismatch and loss of interactions. However, interactions among plant species can also vary with climate [5–8,17]. For instance, a removal experiment in alpine ecosystems found that neighbor interactions shifted from facilitative to competitive with rising mean temperature at a site [7,9–11,18]. Interspecific competition for resources could also influence phenology; for instance shading by overstory species can delay flowering and reduce reproductive success in understory species [5,8,19,20].

As species shift their ranges in response to climate change, novel species interactions (such as shading by newly arriving overstory species) could arise with the potential to alter phenological and performance responses to climate change. In arctic and alpine regions, shrubs could initiate these novel interactions, as many shrub species have been expanding their ranges over time [13,21,22], and increasing in abundance in response to experimental warming [14,15,23,24]. Encroachment by shrubs has the potential to alter the phenology of high-light adapted species. However, to our knowledge, phenological responses to recent colonization of shrubs under conditions of enhanced warming have not been observed.

In California’s White Mountains, *Artemisia rothrockii* (sagebrush) has expanded its range margin upward in elevation over the past half-century during a period when local temperatures increased and precipitation decreased [25]. The subalpine and alpine plant communities in the White Mountains are dominated by cushion plants (prostrate perennial herbs), which have declined in abundance over this same time period [25]. Cushion plants are foundational species that increase species diversity through facilitation in arctic and alpine ecosystems worldwide, and may play important roles in buffering these ecosystems against biodiversity declines in the face of climate change [26]. To evaluate whether cushion plant declines were caused directly by climate change, or indirectly via sagebrush encroachment, we conducted an artificial warming experiment in plots both with and without sagebrush present, at two elevations (see Methods). Additional shading treatments were also imposed to isolate the impact of shading versus other potential impacts of shrubs, such as influences on soil resources. We focused on flowering phenology and flower production responses of *Trifolium andersonii* (Anderson’s clover, S1 Fig) because in our study area it is the most abundant species and the only species common across the elevational range of our study sites.

## Methods

This experiment was carried out in California’s White Mountains under a Special Use Permit administered by the Inyo National Forest. The White Mountains are located on the western edge of North America’s Basin and Range Province and lie within the rain shadow of the Sierra Nevada Mountains and are in a transition zone between the maritime influence of the Pacific Ocean and the continental influence of North America resulting in a cold and arid climate. Winter snow accounts for much of the yearly precipitation, which varies with elevation, ranging from 456 mm/yr at Barcroft Station (3800 m) to 327 mm/yr at Crooked Creek Station (3094 m). Temperature is also strongly dependent on elevation, with a mean annual temperature of -1.7°C at Barcroft Station, and 0.9°C at Crooked Creek Station [27]

The influences of warming and sagebrush encroachment on the alpine plant community were evaluated experimentally at two elevations in the White Mountains: 3700 m (37° 34.1’ N, 118° 14.3’ W), and 3100 m (37° 29.9’ N, 118° 10.3’ W). The study was initiated in late-June, 2011. At each elevation, 0.785-m^2^ plots were established at least 2 m apart on granitic derived soils and arranged in a randomized design with 4 replicate plots of each of the 5 treatments: (1) shaded without *Artemisia rothrockii* (shade), (2) warmed with established *A*. *rothrockii* plants (warmed sagebrush), (3) warmed without established *A*. *rothrockii* plants (warm) and (4) unwarmed controls with established *A*. *rothrockii* plants (sagebrush) and (5) without established *A*. *rothrockii* plants (open) for a total of 20 plots at each elevation. Plots were selected to have comparable starting species composition and cover of commonly shared species at each elevation.

Warming chambers were constructed following the methods developed by the International Tundra Experiment [28] and were made of 5 oz clear Crystalite fiberglass (thickness = 1.1 mm, light transmission = 90%, Ridout Plastics Company Inc., San Diego, CA, USA; S2 Fig). Warming chambers were 1-m diameter and were held in place year round using 152 mm spike nails. Shading structures were constructed with fiberglass window screen and intercepted 60% (as measured with a Decagon Devices AccuPAR LP-80 PAR sensor). The window screen was suspended 20 cm above the soil surface on a PVC frame (S2 Fig).

Air temperatures in each treatment were monitored using iButton temperature loggers (Maxim Integrated Products, Sunnyvale, CA, USA). For these analyses mean growing season temperatures were calculated between May 4 and June 30, 2013. To shield against solar radiation, the temperature loggers were attached to the interior of 5.1 cm diameter by 7.6 cm long white PVC pipe, held in place with landscape staples.

Beginning in early-May, 2013, the third season of experimental warming, flower production of *Trifolium andersonii* was monitored on alternating days at both 3100 m and 3700 m. On each sampling date the number of open flowers was recorded. Percent cover of *T*. *andersonii* in each plot was recorded at the peak of the growing season in late-June and early-July.

Statistical analysis was conducted using the statistical programming language R, version 3.2.1 (R Core Development Team, 2015). The influence of the treatments on air temperature was evaluated with a linear mixed-effect model where warming, elevation and sagebrush presence were included as fixed factors, and plot was included as a random factor to account for repeated measures. Date of first flowering, peak flowering date, and flower production (total flowers produced on peak flowering date) were analyzed using linear models where Warming, Elevation and Sagebrush presence were included as factors, along with all possible interaction terms. Statistical significance was evaluated using type II (marginal) tests. The shade treatment was compared against unwarmed open and sagebrush controls at each elevation via Welch’s Two Sample t-test.

To evaluate whether Sagebrush modified phenological responses to warming via influences on temperature, we calculated phenological sensitivity to warming as days shift in phenology (observed value in warmed plot—mean across control plots) divided by degrees shift in growing season temperature (mean across treatment plots—mean across control plots), as in [8]. Phenological sensitivity to warming was then compared in plots with and without sagebrush present using a linear model where elevation and sagebrush were included as interacting fixed factors.

## Results and Discussion

### Temperature manipulation

The warming chambers increased temperatures 1.8°C in plots at the lower elevation site (F = 14.8, p<0.01), and 2.4°C at the higher elevation site (F = 11.1, p<0.01). These effects were not modified by statistical interactions among factors, and there was no significant impact of sagebrush (69% light interception) presence on plot temperatures (treatment means in Table 1, statistics in S1 Table). At 3100 m, shaded plots were 0.5°C cooler than open control plots (t = 2.20, p = 0.03, S3 Fig; S2b Table), but temperature in shaded plots did not differ from open controls at 3700 m, nor from Sagebrush plots at either elevation.

**Table 1:** Temperature and Phenology Responses to Warming.

			Flowering Day of Year Difference from Control		Flowering Sensitivity (days/°C)	
	Treatment	Temperature Difference (°C) from Control	First Flower	Peak Flower	First Flower Date	Peak Flower Date
**3100 m**	Warmed	2.3 (0.4)	-18.9 (3.3)	-16.0 (4.1)	-4.5 (2.3)	-7.1 (2.2)
	Warmed + Sagebrush	1.9 (0.4)	6.4 (3.3)	-17.8 (4.1)	1.1 (2.3)	-9.5 (2.2)
**3700 m**	Warmed	3.2 (0.5)	6.1 (4.4)	-11.4 (3.1)	-2.1 (2.5)	-3.7 (3.0)
	Warmed + Sagebrush	2.4 (0.5)	19.1 (4.4)	-8.4 (3.1)	2.6 (2.5)	-3.6 (3.0)

Mean responses (with standard error) of temperature, flowering date and flowering sensitivity to warming treatments.

### Phenological response

**Fig 1:**
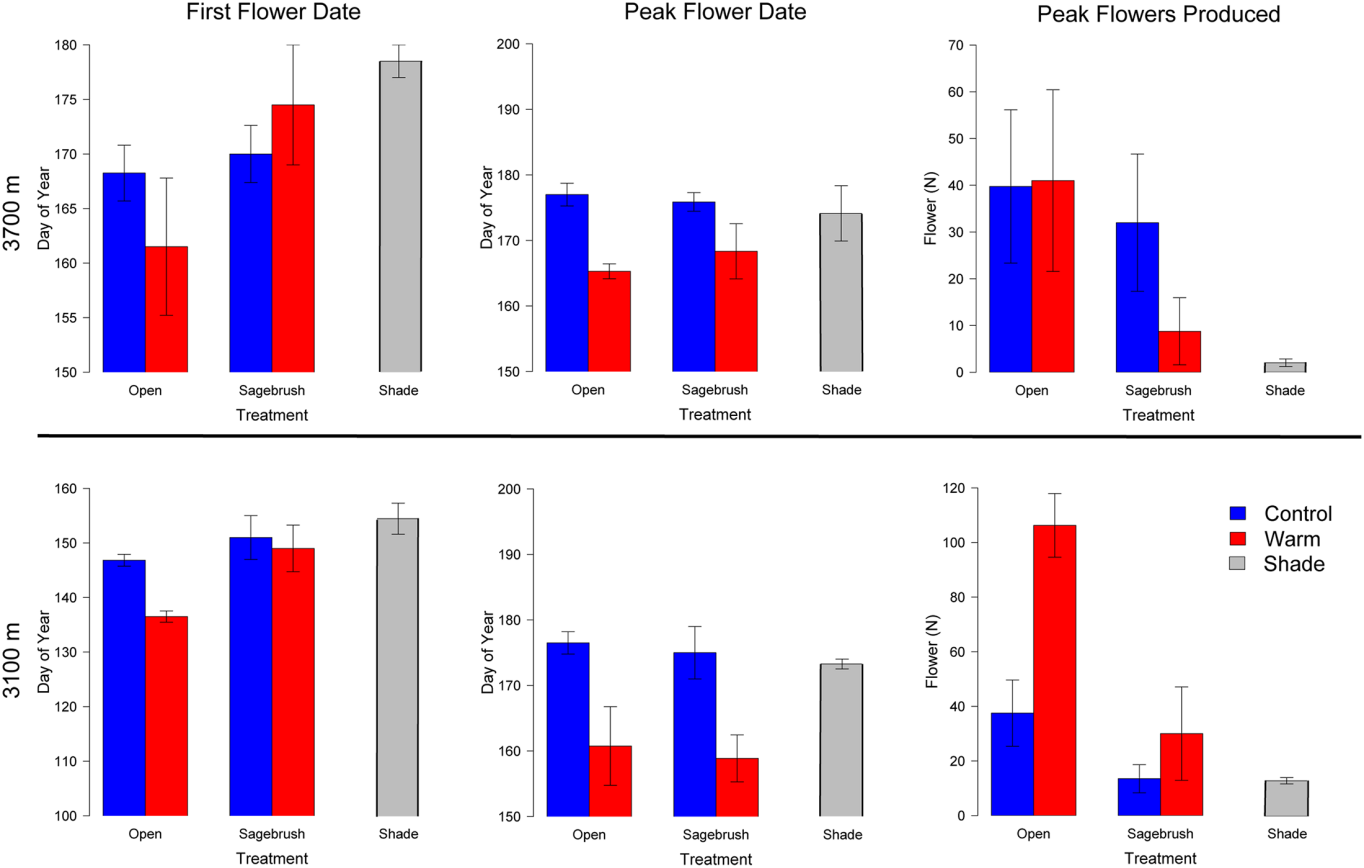
First flower date (A, D), peak flower date (B, E) and peak flowers produced (C, F) for *Trifolium andersonii* at 3700 m (A-C) and 3100 m (D-F). Shade and open treatments contained no sagebrush and shade treatments were not warmed.

As expected, *T*. *andersonii* flowered earlier in response to warming (F_1,23_ = 9.58, p<0.01). The presence of sagebrush, however, delayed first flowering date (F_1,23_ = 10.3, p<0.01), and modified the phenological response of *T*. *andersonii* to warming (warming*sagebrush F_1,23_ = 4.63, p = 0.04). In the absence of sagebrush, warming advanced flowering phenology of *T*. *andersonii* by -4.5 days per °C warming at 3100 m (-2.1 days per °C of warming at 3700 m; Fig 1; Table 1; S1a Table). This magnitude of phenological advancement is consistent with, and even exceeds, other passive warming experiments in arctic and alpine systems that have found advances in the onset of flowering dates in the range of -1.9–3.3 days per °C of warming [29]. The presence of sagebrush, however, delayed first flowering of *T*. *andersonii* in both warmed and unwarmed plots compared to controls (+1.1 days per °C at 3100 m and +2.6 days at 3700 m; Fig 1; Table 1; S1a Table), demonstrating that species interactions can influence phenological responses to climate change. There was a similar pattern for peak flowering dates, which were significantly advanced in warmed open and warmed sagebrush treatments at both elevations (F_2,26_ = 31.6, p<0.01; Fig 1; S1a Table).

Multiple lines of evidence support shading as a potential mechanism by which sagebrush alters *T*. *andersonii*’s phenological response to warming. First, sagebrush presence had no significant influences on plot temperature as previously described. In addition, first flowering date was later in shaded compared to open plots (approximately +7.7 days at 3100 m and +10.3 days at 3700m, t = -3.00, p = 0.03; Fig 1; S1b Table). There was also a significant difference in the sensitivity of *T*. *andersonii* first-flowering to warming between plots with and without sagebrush (F_1,12_ = 6.32, p = 0.03, Table 1) suggesting that something other than temperature was responsible. For peak flowering, however, there was no significant difference in sensitivity to warming between plots with and without sagebrush (F_1,12_ = 0.66, p = 0.43). Hence, the sensitivity of flowering phenology to rising temperatures may differ over the course of the growing season or may be non-linear (resulting in a significant change in phenology despite non-significant changes in temperature). Alternatively, the phenology of peak flowering may have been altered via the overall suppression of flower production by sagebrush as described below.

### Flower production

In the absence of sagebrush, warming strongly enhanced total flower production at 3100 m (warming x elevation; F_1,26_ = 7.66, p = 0.01; Fig 1; S1a Table). However, there was a strong negative effect of sagebrush presence on total flower production (sagebrush; F_1,26_ = 12.5, p<0.01; Fig 1; S1a Table). Despite minimal effects on temperature, artificial shading delayed phenology and suppressed peak flower production at both elevations (Fig 1; S1b Table) supporting the idea that shading by sagebrush likely influences understory plants both directly through light limitation to growth, and also indirectly by modifying phenological cues associated with light.

### Implications for altered species interactions

Species interactions play critical roles in determining plant community composition, and shifts in the strength or direction of species interactions could dramatically influence plant population and community responses to future climate change [17]. For instance, removal experiments in a large study across 11 mountain ranges showed that alpine species interactions shifted from mostly facilitative at high elevations and cold sites, to more competitive at low elevations and warmer sites [18]. Similarly, experimental studies manipulating the timing of snowmelt in tundra [30] have found a shift from facilitative to competitive interactions associated with a transition from more stressful to less stressful early season conditions.

Our findings demonstrate that shade-producing species could substantially modify phenological responses to rising temperatures if they establish in communities containing species adapted to high light. The delay of flowering due to shading in this system differs from patterns in deciduous forests, where understory species phenology is accelerated by shade, likely an evolutionary adaptation enabling maximum carbon gain prior to canopy closure [31]. Many alpine and arctic tundra species rely on light cues related to snowmelt to initiate growth and flowering [32,33], hence shading may alter phenological sensitivity to warming for many species with light-based phenological cues. Shading by overstory species could influence community composition via other influences on regeneration; for instance overstory shading increased seedling survival during a heat wave in mesic sites, while exacerbating seedling mortality in dry sites where overstory species competed for limiting soil moisture [34].

Prior studies have proposed that “phenological tracking” of climate enables species to maintain fitness in the face of climate change [35]. Consistent with this hypothesis, in this short-term experiment *T*. *andersonii* had both earlier flowering phenology and greater flower production in warmed plots (Fig 1; S1 Table). Over the last 50 years, however, the abundance of *T*. *andersonii* has declined steeply at this study site during a time of both rising temperatures and increasing encroachment by sagebrush [25], suggesting that the long-term declines may have been caused by an indirect effect of warming on encroachment by sagebrush, rather than a direct response to warming itself. Further, cover of this species has declined rapidly at lower elevations since the establishment of this experiment in 2011, with the greatest declines occurring in warmed treatments where sagebrush was present (S4 Fig). These results demonstrate how altered species interactions could contribute to the large variation in phenological sensitivities to rising temperatures documented in the literature (e.g. [5,6,8]). Furthermore, shifts in species composition may underlie the divergent results of short-term warming experiments and observations made over time in a warming climate [8], because range shifts initiated by rising temperatures may require years or even decades [36] much longer than the duration of most experiments.

Shifting species composition can cause feedbacks to populations, communities and ecosystems responding to climate change [36], an important class of indirect effects. While there are previously documented examples of strong indirect effects of climate change arising via altered biotic interactions [17,30,34,37–39], this study demonstrates for the first time that indirect effects can arise via inhibition: a range expansion by an overstory plant species altered the phenological response to warming by shaded understory species. These findings demonstrate the importance of shifts in species composition, particularly shrub encroachment, in mediating indirect effects of climate change on plant communities.

## Supporting Information

S1 Fig *Trifolium andersonii* is a low growing cushion plant that has experienced declines in abundance during the past half-century in California’s White Mountains. (TIFF)

S2 Fig Warming chamber (foreground) and shading structures at the 3700 m site in California’s White Mountains. (TIFF)

S3 Fig Mean temperature response to treatments at 3700 m (top) and 3100 m (bottom). Shade and open treatments contained no sagebrush and shade treatments were not warmed.
(TIFF)

S4 Fig Total cover of *Trifolium andersonii* at 3700 m (top) and 3100 m (bottom). Shade and open treatments contained no sagebrush and shade treatments were not warmed.
(TIFF)

S1 Table (a) Statistical analysis of *Trifolium andersonii* phenology to warming and sagebrush presence across elevations. Three metrics were analyzed: date of first flower, date of peak flowering and maximum flowers. (b) Post-hoc analysis of differences in phenology between open, shaded, and sagebrush plots, to evaluate whether shading was a mechanism that could explain the impact of sagebrush on *Trifolium andersonii* phenology.
(PDF)

S2 Table (a) Statistical analysis of temperature in response to warming and sagebrush presence across elevations. (b) Comparative responses of temperature to shading, shade and sagebrush at 3100 m and 3700 m.
(PDF)
