# Supplementary figures and images for: A Range-Expanding Shrub Species Alters Plant Phenological Response to Experimental Warming
Source: PLoS One. 2015 Sep 24;10(9):e0139029. doi: 10.1371/journal.pone.0139029 (PMC4581864; doi:10.1371/journal.pone.0139029)

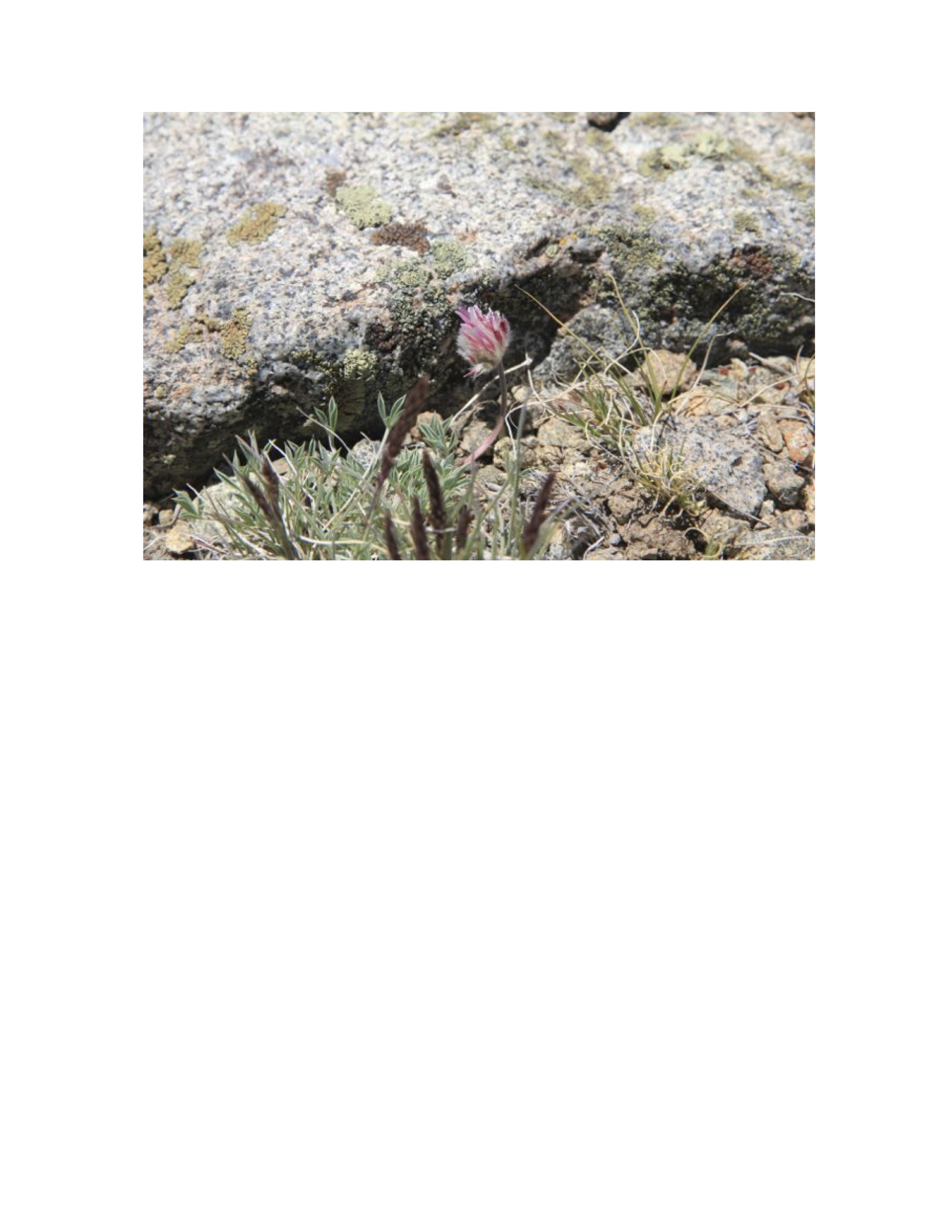

Supplement: S1 Fig — (TIFF) [file pone.0139029.s001.tiff]

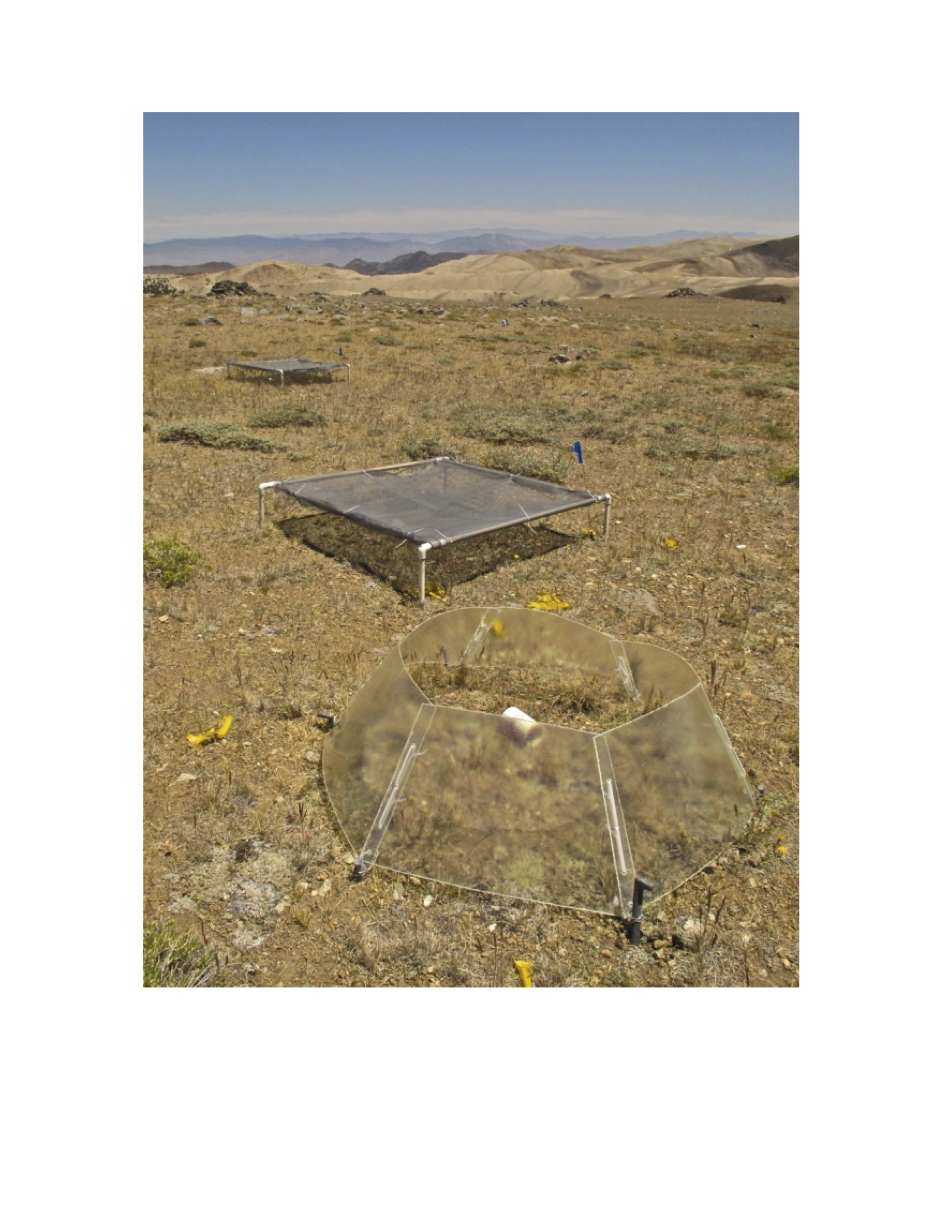

Supplement: S2 Fig — (TIFF) [file pone.0139029.s002.tiff]

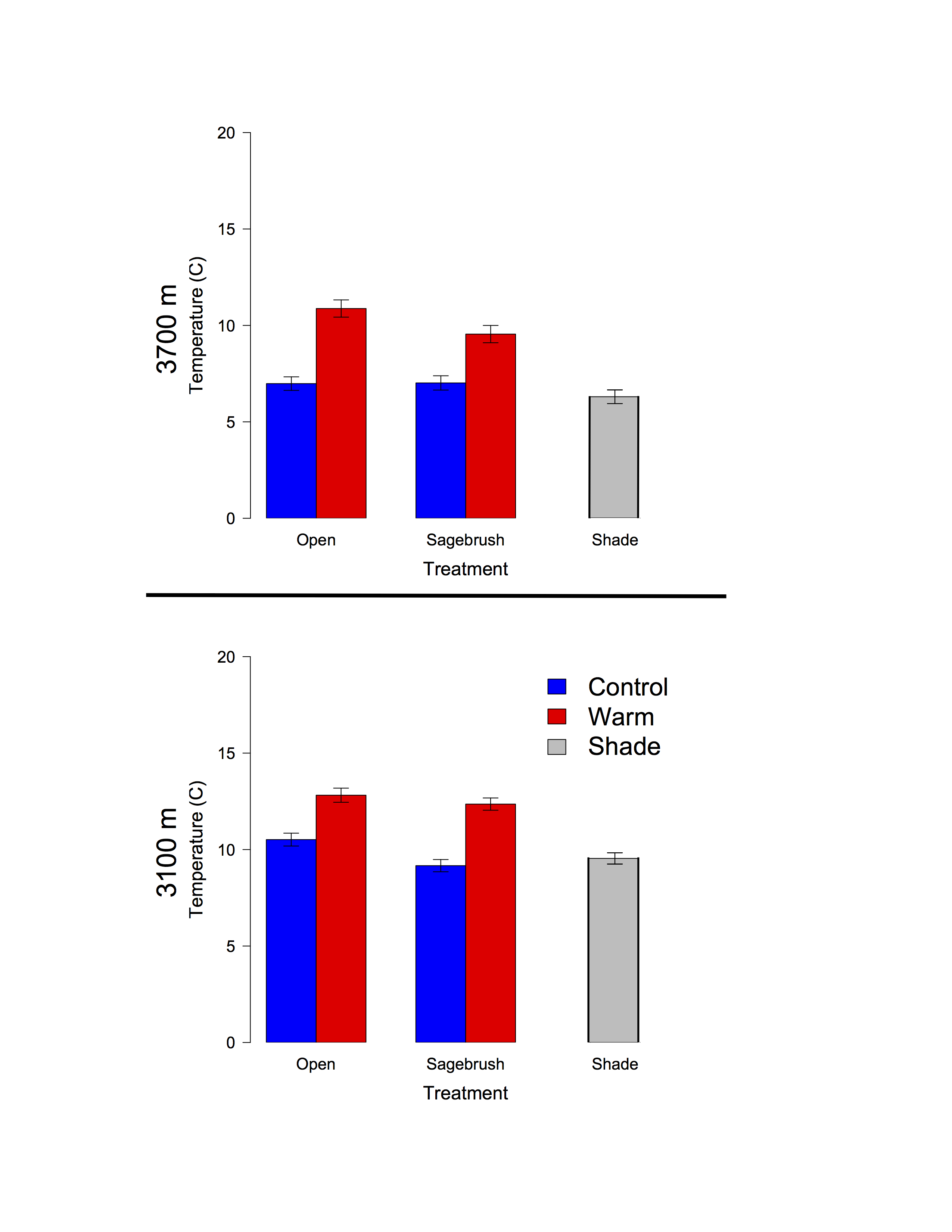

Supplement: S3 Fig — Shade and open treatments contained no sagebrush and shade treatments were not warmed. (TIFF) [file pone.0139029.s003.tiff]

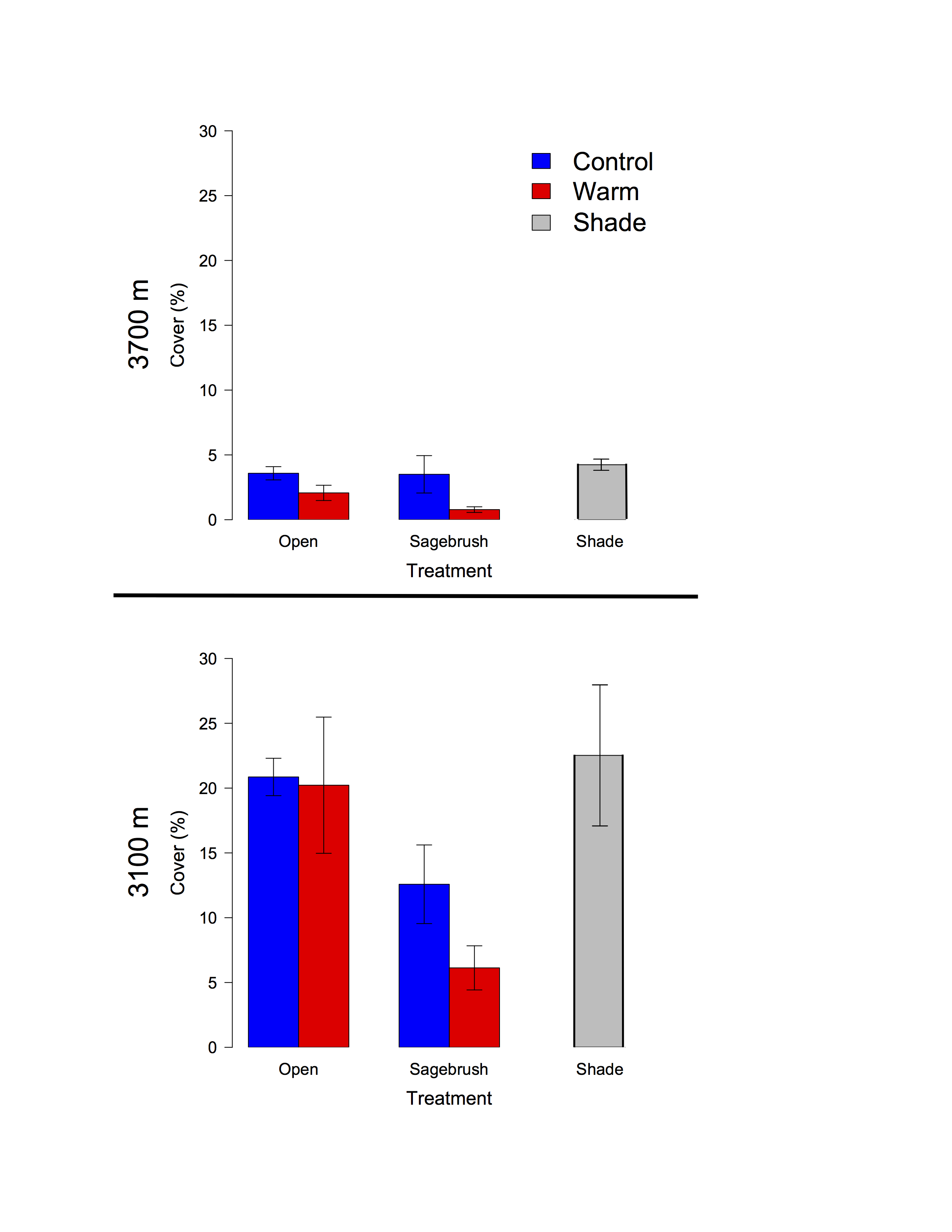

Supplement: S4 Fig — Shade and open treatments contained no sagebrush and shade treatments were not warmed. (TIFF) [file pone.0139029.s004.tiff]
