# Supplementary material for: A Range-Expanding Shrub Species Alters Plant Phenological Response to Experimental Warming
Source: PLoS One. 2015 Sep 24;10(9):e0139029. doi: 10.1371/journal.pone.0139029 (PMC4581864; doi:10.1371/journal.pone.0139029)
Supplement: S1 Table — (a) Statistical analysis of Trifolium andersonii phenology to warming and sagebrush presence across elevations. Three metrics were analyzed: date of first flower, date of peak flowering and maximum flowers. (b) Post-hoc analysis of differences in phenology between open, shaded, and sagebrush plots, to evaluate whether shading was a mechanism that could explain the impact of sagebrush on Trifolium andersonii phenology. (PDF) [file pone.0139029.s005.pdf]

**a.**	DOY First Flower		DOY Peak Flowering		Maximum Flowers	
	$F_{1,23}$	p	$F_{1,26}$	p	$F_{1,26}$	p
Elevation	77.1	**<0.01**	2.58	0.12	2.53	0.12
Warming	9.58	**0.01**	31.6	**<0.01**	3.52	0.07
Sagebrush	10.4	**<0.01**	0.03	0.86	12.5	**<0.01**
Elevation*Warming	0.05	0.83	1.83	0.19	7.66	**0.01**
Elevation*Sagebrush	0.04	0.84	0.31	0.58	2.27	0.14
Warming*Sagebrush	4.61	**0.04**	0.15	0.70	3.96	0.06
Elevation*Warming*Sagebrush	0.15	0.70	0.24	0.63	0.50	0.49

**b.**	Shade vs. Open		Shade vs. Sagebrush	
	t	p	t	p
First Flower DOY (3100 m)	-2.52	0.07	-0.71	0.51
First Flower DOY (3700 m)	-3.00	**0.03**	-2.43	0.06
Peak Flowering DOY (3100 m)	1.74	0.13	0.43	0.69
Peak Flowering DOY (3700 m)	0.65	0.55	0.41	0.70
Maximum Flowers (3100 m)	2.00	0.10	0.09	0.93
Maximum Flowers (3700 m)	2.30	0.10	2.04	0.13
