# Supplementary material for: A Range-Expanding Shrub Species Alters Plant Phenological Response to Experimental Warming
Source: PLoS One. 2015 Sep 24;10(9):e0139029. doi: 10.1371/journal.pone.0139029 (PMC4581864; doi:10.1371/journal.pone.0139029)
Supplement: S2 Table — (a) Statistical analysis of temperature in response to warming and sagebrush presence across elevations. (b) Comparative responses of temperature to shading, shade and sagebrush at 3100 m and 3700 m. (PDF) [file pone.0139029.s006.pdf]

**a.**	DF	*F*	*p*
Elevation	1,50	14.8	**<0.01**
Warming	1,50	11.1	**<0.01**
Sagebrush	1,50	1.00	0.32
Elevation*Warming	1,50	0.004	0.95
Elevation*Sagebrush	1,50	3.08	0.09
Warming*Sagebrush	1,50	0.00	1.00
Elevation*Warming*Sagebrush	1,50	3.26	0.08

**b.**	Shade vs. Open		Shade vs. Sagebrush	
	t	*p*	*t*	*p*
3100 m	2.20	**0.03**	-0.87	0.38
3700 m	1.40	0.16	1.43	0.15
